# Unique anti-angiogenic effects, pharmacological targets and therapeutic mechanisms of Chinese herbal medicines for endometriosis

**DOI:** 10.1016/j.gendis.2023.101166

**Published:** 2023-11-11

**Authors:** Bo Liang, Rui Dong, Sze Wan Hung, Yiran Li, Yuezhen Lin, Ling Wu, Tao Zhang, Gene Chi Wai Man, Hui Xu, Jacqueline Pui Wah Chung, Chi Chiu Wang

**Affiliations:** a Guangdong Provincial Key Laboratory of Marine Biotechnology, Institute of Marine Sciences, Shantou University, Shantou, Guangdong 515063, China; b Department of Obstetrics and Gynaecology, The Chinese University of Hong Kong, Hong Kong SAR, China; c Reproduction and Development Laboratory, Li Ka Shing Institute of Health Sciences, The Chinese University of Hong Kong, Hong Kong SAR, China; d School of Biomedical Sciences, The Chinese University of Hong Kong, Hong Kong SAR, China; e Chinese University of Hong Kong-Sichuan University Joint Laboratory in Reproductive Medicine, The Chinese University of Hong Kong, Hong Kong SAR, China

Endometriosis is a common and benign angiogenesis-dependent gynecological disorder, which refers to the proliferation and growth of endometrium-like tissues with neovasculature formation outside of the uterus.^1^ Available medical treatments for endometriosis containing hormonal and non-hormonal treatments had been limited for long-term usage by their side effects.^2^ Ideal medical treatment for endometriosis with efficacy to relieve symptoms and suppress endometriotic lesion growth and minimal side effects has been longing for decades.^3^ Angiogenesis is a promising therapeutic target for endometriosis.^4^ Chinese herbal medicines (CHM), as a mainstream medication in China and many other Asian countries, have been commonly used for women with endometriosis.^5^ However, there is no scientific evaluation of their anti-endometriosis and anti-angiogenic effects on endometriosis. Clinical trials can only include limited interventions for comparison and a large sample size is required to achieve statistical power for outcome measures of interest. Herewith, an experimental endometriosis mouse model was applied to investigate and compare the anti-angiogenic effect, targets, and mechanism of CHM. In this study, anti-angiogenic effects, pharmacological targets, and therapeutic mechanisms of commonly used CHM formulae, including Shaofu Zhuyu Tang (SFZY), Xuefu Zhuyu Tang (XFZY), Gexia Zhuyu Tang (GXZY), Wenjing Tang (WJD), Taohe Chengqi Tang (THCQ), and Taohong Siwu Tang (THSW) in the mouse model were studied (Table S1).

The establishment of the endometriosis model and intervention was performed as described in supplementary methods and materials. After intervention, we measured endometriotic lesion size and weight. In contrast with vehicle control, CHM formulae XFZY, THCQ, THSW, and positive control dienogest significantly reduced the endometriotic lesion size up to 42.4%, 48.4%, 48.5%, and 38.9%, respectively, and lesion weight up to 46.9%, 48.8%, 47.4%, and 37.7%, respectively (Fig. 1A). No significant differences in the endometriotic lesion size and weight were found between SFZY, GXZY, and WJD groups. The cyst-like endometriotic lesions were underdeveloped in XFZY, THCQ, and THSW groups (Fig. 1B, C). Immunohistochemistry staining was performed to confirm the expression of vascular endothelial growth factor (VEGF) after treatment with XFZY, THCQ, and THSW. As shown in Figure 1D and 1E, decreased VEGF expression in both epithelial and stroma cells of the endometriotic lesions was found after treatment with XFZY and THCQ. In addition, anti-proliferative and pro-apoptotic effects of effective CHM formulae were evaluated with immunohistochemistry staining. Ki67 positively stained proliferating cells in both epithelial and stroma cells in the endometriotic lesions of XFZY, THCQ, and THSW groups were decreased but no significant difference was found (Fig. S1B–D). TUNEL positively stained apoptotic cells in the epithelial and stroma cells of the endometriotic lesions were increased in XFZY group (Fig. S1F–H).

**Figure 1:**
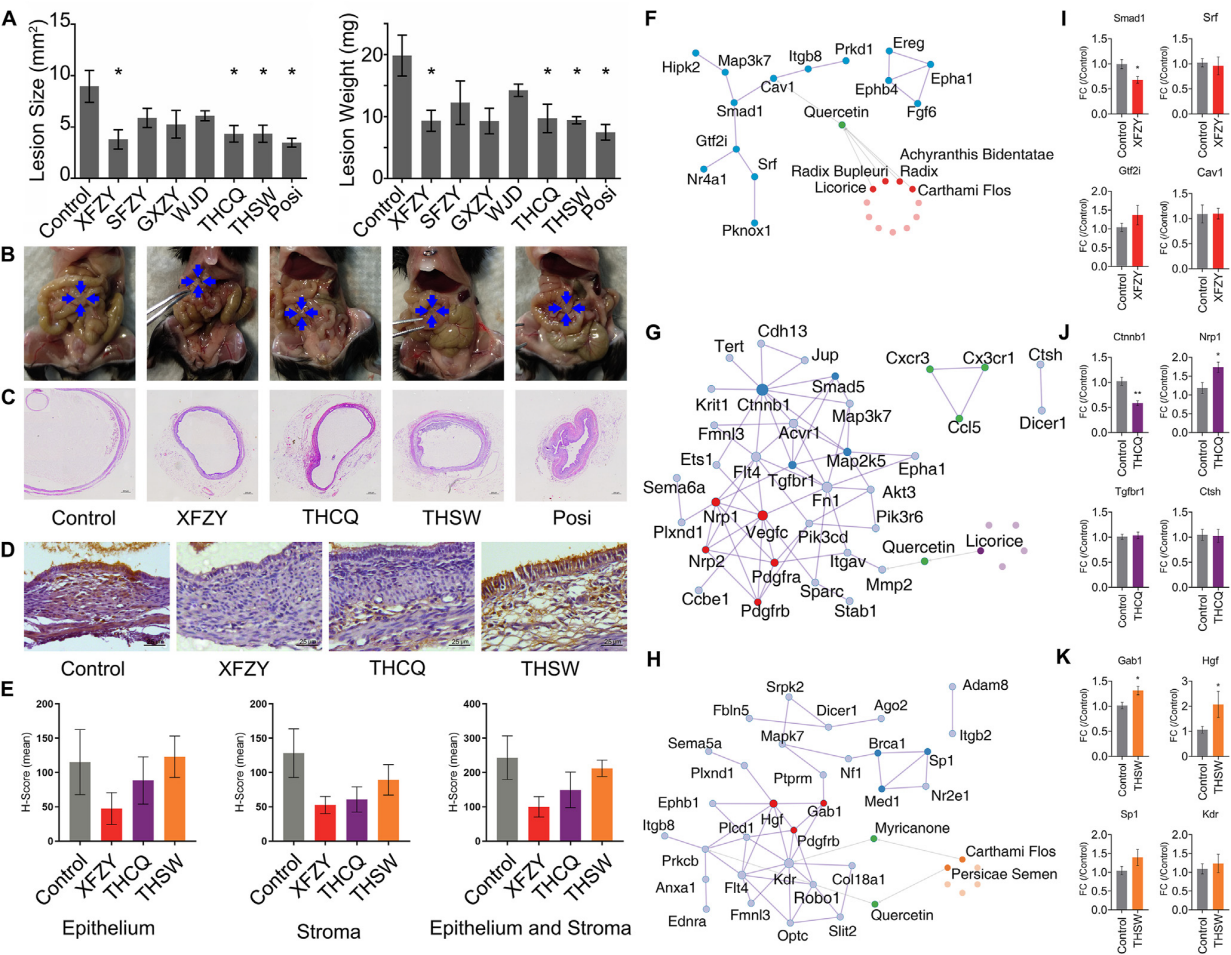
Efficacy, anti-angiogenic effects, and potential therapeutic mechanisms of Chinese herbal medicines (CHM) for endometriosis. **(A)** The efficacy assessment of CHM formulae on endometriotic lesion development in a mouse model by endometriotic lesion size and lesion weight. Data were shown as mean ± standard error of the mean (SEM). *n* = 4 for control group and *n* = 3 for each CHM formula group; ^∗^*P* < 0.05 *vs*. the negative control group. **(B)** Morphology of endometriotic lesions after treatment with negative control or a CHM formula. **(C)** Histological change of endometriotic lesions after treatment. **(D)** Representative images of VEGF staining in endometriotic lesions. Scar bar = 25 μm. **(E)** Staining score of VEGF in different sections of ectopic lesions in five random views. Data were shown as mean ± SEM. *n* = 4 for control group and *n* = 3 for each CHM formula group; ^∗^*P* < 0.05 *vs*. the negative control group. **(F–H)** Key protein–protein interaction networks of differential expression angiogenic genes from three formulae. **(I–K)** Expression levels of key genes identified from key protein–protein interaction networks of differential expression angiogenic genes from three formulae. ^∗^*P* < 0.05 *vs*. the negative control group.

To identify the potential therapeutic targets and mechanisms of the effective CHM formulae, gene expression was measured by the Agilent microarray platform (Agilent Technologies, CA, USA). Differentially expressed genes with *P* values < 0.01, |log2FC| ≥ 2, and adjusted *P* value < 0.05 were considered statistically significant. Gene expression profiling and functional clustering analysis with differentially expressed genes were performed as described in supplementary methods and materials and additional results (Fig. S2, 3). Potential targets of active components from CHM were revealed from the Traditional Chinese Medicine Systems Pharmacology (TCMSP) database and analysis platform.

Although all three formulae disrupted angiogenesis in endometriotic lesions, their molecular interaction networks and dysregulated expression of angiogenesis-related differential expressed genes were different (Fig. 1F, G, K). Potential interaction between active components (β-carotene and quercetin) and target gene (Cav1) was found in XFZY and the mRNA expression level of Smad1 was significantly suppressed while Gtf2i was up-regulated by XFZY (Fig. 1I), indicating that XFZY may suppress the expression level of angiogenic factor VEGF via suppressing Smad1 expression. Only the active component quercetin was found to interact with Mmp2 in THCQ (Fig. 1G) and THCQ suppressed expression of Ctnnb1 and up-regulated Nrp1 (Fig. 1J), suggesting a distinct anti-angiogenic effect of THCQ when compared with XFZY. Degradation of extracellular membranes through Mmp2 and suppression of VEGF signaling by THCQ required further study. Two active components of THSW (myricanone and quercetin) were found interacting with Pdgfrb and Kdr and THSW up-regulated expression of Gab1, Hgf, and Sp1 (Fig. 1K), suggesting that THSW may pose anti-angiogenic effect to endometriotic lesions by both down-regulating VEGF expression and interacting with receptors Pdfgrb and Kdr (Fig. 1G, J). Thus, our results suggested that CHM formulae XFZY, THCQ, and THSW suppressed the angiogenesis of endometriotic lesions via diverse pharmacological targets and underlying molecular mechanisms, which may be due to the different compositions of individual herbs. The safety of the CHM formula on endometriosis mice was also analyzed. No significant changes in body weight and uterine and ovary size and weight were found after treatment of different CHM formulae. No obvious histological changes in the uterus (Fig. S4D, upper panel) and ovary (Fig. S4D, lower panel) were found. However, CHM formula XFZY significantly decreased uterine gland count in endometrium and antral follicle count in the ovary (Fig. S4E, G) but with no significant change in endometrium thickness index (Fig. S4F). An increase in uterine gland count was found after treatment with dienogest (Fig. S4E).

In conclusion, we demonstrated that CHM formulae XFZY, THCQ, and THSW significantly suppressed endometriotic lesion growth and development with a good safety margin in an experimental endometriosis model in mice. Moreover, distinct and unique therapeutic mechanisms of each CHM formula were identified and their potential active components and pharmacological targets were found. However, their pharmacological profile and active components are still unknown. Further studies are needed to confirm the potential of CHM for the treatment of endometriosis.

## Conflict of interests

The authors declare no conflict of interests.

## Funding

This study was partially supported by the Strategic Seed Funding for Collaborative Research Scheme (SSFCRS) of the Chinese University of Hong Kong (CUHK, China) (No. 0670/22) and the National Natural Science Foundation of China (No. 82260948).

## Appendix A Supplementary data

Multimedia component 1 Multimedia component 1

Fig s1 Fig s1

Fig s2 Fig s2

Fig s3 Fig s3

Fig s4 Fig s4
