# Supplementary material for: Unique anti-angiogenic effects, pharmacological targets and therapeutic mechanisms of Chinese herbal medicines for endometriosis
Source: Genes Dis. 2023 Nov 11;11(5):101166. doi: 10.1016/j.gendis.2023.101166 (PMC11177055; doi:10.1016/j.gendis.2023.101166)
Supplement: Multimedia component 1 [file mmc1.docx]

Supplementary data for

**Unique anti-angiogenic effects, pharmacological targets and therapeutic mechanisms of Chinese herbal medicines for endometriosis**

Bo Liang^1,2,#^, Rui Dong ^1,#^, Sze Wan Hung^2^, Yiran Li^2^, Yuezhen Lin^2^, Ling Wu^2^, Tao Zhang^2^, Gene Chi Wai Man^2^, Hui Xu^2^, Jacqueline Pui Wah Chung^2^, Chi Chiu Wang^2,3,4,5,*^.

#: co-first author

*: corresponding author

Corresponding address: Dr. Chi Chiu Wang, Department of Obstetrics and Gynaecology, The Chinese University of Hong Kong, c/o 1st Floor, Special Block E, Prince of Wales Hospital, Shatin, Hong Kong. ccwang@cuhk.edu.hk.

**This file includes:**

**Additional results**

**Methods and Materials**

**Supplementary Fig. S1 to S7**

**Supplementary Table. S1**

**Additional results**

**Anti-Proliferative effect and apoptotic effect of effective CHM formulae.**

Ki67 positively stained proliferating cells in both epithelial cells and stroma cells in the endometriotic lesions of XFZY, THCQ and THSW groups were decreased (Fig S1. A-D). TUNEL positively stained apoptotic cells in the epithelial cells and stroma cells of endometriotic lesions were increased in XFZY, THCQ and THSW groups (Fig S1. E-H).

**CHM therapeutic actions on endometriosis**

Enrichment analysis of differential expressed genes (DEGs) after treatment with XFZY were significantly enriched in actomyosin structure organization and regulation of cell projection organization, adherent junction, monnose type O-glycan biosynthesis and aldosterone synthesis and secretion (Fig S2. A). And network pharmacological analysis indicating active components including Isorhamnetin, Quercetin and Formononetin as the key CHM ingredients triggered these signaling pathways (Fig S2. E). According to the functional enrichment analysis for THCQ, we also found that Adherens junction, Focal adhesion and Lysosome were affected based on Kegg database (Fig S2. B). As for network pharmacological analysis, Aloe-emodin from CHM Da Huang (Radix Rhei Et Rhizome) link to PI3K-Akt, MAPK, JAK-STAT, T cell receptor and Apelin signaling pathways (Fig S2. F). Lysosome organization and Choline metabolism were the significantly enriched biological process in THSW treatment (Fig S2. C), and network pharmacological analysis indicated Luteolin, Quercetin, Kaempferol and Myricanone of Hong Hua (Carthami Flos), Bai Shao (Paeoniae Radix Alba) and Chuan Xiong (Paeoniae Radix Alba) as the key CHM ingredients link to Lysosome formation, Ras and Phosphatidylinositol signaling pathways and Choline metabolism (Fig S2.C and F).

**Anti-angiogenesis mechanism on endometriosis**

XFZY, THCQ and THSW had 33, 47 and 49 differential expressed angiogenesis genes and there are only 22 common genes between 2 CHM but none amongst all 3 CHM (Fig S3. A, J). DEGs of all 3 formulae were clustered to biological process including: inflammatory response, cell-cell adhesion, regulation of cell adhesion, epithelial cell proliferation and regulation of angiogenesis (Fig S3. B to E). For XFZY, Vegfc expression was positively correlated with Catsper2, Ushbp1, Fam205c, Syde1, Cyp4f39, and Trim56 and negatively correlated with Slc25a47, Acadvl and Fstl3. For THCQ, Vegfc expression was positively correlated with Fut9, Kif21a, Pnrc1, Phf3 and Zzef1 and negatively correlated with Kansl1, Mettl6 and Runx3. Even though significant correlation between Vegfc and other DEGs after treatment with THSW was not found, Pdgfrb was negatively correlated with B4gat1 (Fig S3. F to H)**.**

**Safety of CHM formula and individual herbs on endometriosis mice**

As shown in figure s4 A-C, no significant changes in body weight, uterine and ovary size and weight were found after treatment of different CHM formulae. No obvious histological changes in uterus (Fig S4. D, upper panel) and ovary (Fig S4. D, lower panel) were found. However, CHM formula XFZY significantly decreased uterine gland count in endometrium and antral follicle count in ovary (Fig S4. E and G) but with no significant change in ETI (Fig S4. F). Increase of uterine gland count was found after treatment with DIenogest (Fig S4. E).

**Methods and Materials**

**Materials**

CHM formulae were purchased as concentrated granules manufactured under GMP standard from Nong’s Chinese Medicine Pharmaceutical, Hong Kong. Rabbit monoclonal Ki67antibody were purchased from Cell Signaling Technology (USA) and mouse monoclonal VEGF antibody was purchased from Abcam (USA). *In situ* apoptosis detection kit for TUNEL assay was purchased from Merck Millipore (USA).

**Animals**

C57BL/6 adult female mice aged from six to eight weeks with 18-20g were used in this study. Mice were housed in pathogen-free animal facility with fixed 12 hours’ light and 12 hours’ dark cycle and well-controlled temperature and humidity. Free water and rodent chew were provided. All the procedures in this study were approved by Animal Experimentation Ethics Committee of The Chinese University of Hong Kong.

**Endometriosis model**

Endometriosis model was established as mentioned in previous studies with some modifications ^1;2^. Briefly, after acclimatized for one week, mice were randomly divided into donor group and recipient group in ratio of 1 to 2. Donor mice were sacrificed, and their uterus were dissected and washed in PBS for 3 times. Both horns of uterus were removed and opened by longitudinal incision. Then endometrial tissues were prepared using 2-mm biopsy punch. The endometrial tissues were kept in PBS on ice prior to transplantation. For recipient mice, a 5-mm incision was made on the midline of abdomen wall under anesthetics and then mesentery was pulled outside the peritoneal cavity with blunt forceps. Three pieces of the endometrial tissues were transplanted to mesentery by suturing with a 6-0 nylon surgical thread. Mesentery was then carefully cleaned with PBS with Penicillin and Streptomycin and gently put back into peritoneal cavity with blunt forceps. Abdomen wall and skin were then closed separately by suturing with 5-0 and 4-0 nylon surgical thread.

**CHM formulae selection**

Based on the top 10 commonly used CHM formulae, 6 with therapeutic effect of removing or breaking the “Blood Stasis” were selected for study, namely Xuefu Zhuyu Tang (XFZY), Shaofu Zhuyu Tang (SFZY), Wenjing Tang (WJD), Gexia Zhuyu Tang (GXZY), Taohe Chengqi Tang (THCQ), and Taohong Siwu Tang (THSW) (Table 1). Animal dose of each individual herb in these CHM formulae were calculated based on the clinical dose recommended in Chinese pharmacopoeia ^3;4^ or classical Chinese medicine textbook according to the FDA calculator ^5^

**Interventions**

One week after the endometrium transplantation, all the recipient mice were randomly divided into 8 groups, including negative control group (water as vehicle), positive control group (Dienogest at 0.3mg/kg) and CHM formula groups (XFZY, SFZY, GXZY, WJD, THCQ and THSW). The CHM decoction were freshly prepared by dissolving the granule into appropriated amount of 100ul distilled water and then were administered orally by gavage twice every day for 3 weeks. During the intervention, health and behavior of mice were monitored every day and bodyweight was recorded every five days.

**Sample collection**

All the mice were sacrificed and the endometriotic lesions, uterus and ovary were removed and weighted at the end of intervention. Lesion size was estimated by measuring the longest length (*L*) and perpendicular width (*W*) with a caliper, and was calculated as Lesion size = *L*W**π/4 ^6^. Uterus size was estimated by measuring with a caliper directly. Ovaries were considered as ellipsoid and the ovary size was calculated as 1/6 π × length × width × thickness (mm^3^) according to previous study ^7^.

**H&E staining**

All the endometriosis lesions, ovaries and uteruses were fixed, processed, and embedded for serial sectioning in 4 µm thickness. Every 10 sections were selected and examined under microscope after standard H&E staining according to the previous study ^8^.

**Uterine gland count and endometrium thickness index**

Uterine sections were scanned and analyzed under 100x magnification. All the endometrial glands on each section were labelled and counted by Image J ^9^, then average numbers of gland count from 6 serial sections in every 10 intervals was taken for statistical analysis. Endometrium thickness index (ETI) was evaluated as described before with modification^10^. Briefly, total cross-sectional areas of myometrium (Am), endometrium (Ae) and uterus cavity (Auc) were measured by Image J, according to previous study ^9^. ETI was then calculated using the formula $ETI=\sqrt{\frac{A_{e}}{\pi}}-\sqrt{\frac{A_{uc}}{\pi}}$

**Immunohistochemistry staining and TUNEL assay**

Proliferation in endometriotic lesions was evaluated by immunohistochemical staining with monoclonal rabbit anti mouse Ki-67 antibody (D3B5, CST), while anti-angiogenic effect of CHM was evaluated by immunohistochemical staining with mouse monoclonal Vegf antibody (ab1316, Abcam). After de-paraffin and rehydration, antigen retriever procedure was performed with sodium citrate buffer in microwave for 20 minutes and then incubated with primary antibody for 15 hours, then the sections were incubated with secondary antibody conjugated HRP enzyme (Donkey anti rabbit, Santa Cruz) for 1 hour in room temperature following by color development with DAB kit (k3468, Dako). Apoptotic cells in the lesion were examined using an *in situ* apoptosis detection kit (ApopTAG®, S7100, Millipore, USA) according to the manufacturer’s instruction. Ki67 and TUNEL positively stained cell densities were determined in blind by two different observers, the average of the counts was taken. Both Intra- or inter-observer variabilities are within 5–10%. H-score of Vegf staining was quantified with QuPath as described in previous studies^11^.

**RNA extraction and transcriptomic analysis**

To investigate underlying anti-endometriotic mechanism of the CHM formulae, total RNA was isolated from the endometriotic lesions using QIAGEN RNeasy Mini Kit (QIAGEN, Germany), concentration and quality of the extracted total RNA were examined by Nanodrop spectrophotometry (Wilmington, USA). Gene expression profiling were conducted in Agilent microarray platform (Agilent Technologies, CA). The microarray chips contained whole mouse genome oligonucleotides in 4×44K v2 format (Agilent). Labeling reactions were performed in 1µg total RNA with Cy5-CTP from Agilent Low RNA Input Linear Amplification and Labeling Kit PLUS (Agilent) according to the manufacturer’s protocol. After hybridisation at 65^o^C for 17hrs, the microarray chip was scanned by Agilent Microarray Scanner G2565BA (Agilent). Data was acquired by Feature Extraction and analysed by GeneSpring GX software (Agilent). The signal intensity data were background subtracted, control spot calibrated, and intra-array Lowess normalized. Relative signal intensities and logarithmic ratios were calculated and compared. To calculate significant differential expressed genes (DEGs), one-way ANOVA and pairwise bootstrap statistics were performed to identify significantly expressed genes with p values <0.01. Only Log2FC≥2 and adjust p value <0.05 were considered as statistically significant.

**Network Pharmacology**

To identify the major therapeutic functions of the effective CHM formulae, PPI network was constructed with DEGs found. Interaction of different proteins was extracted from String database^12^. Protein interaction of activation, binding, catalysis, expression, inhibition, post-transcriptional modification was included. Potential target of active components from Chinese herb medicines was extract from Traditional Chinese Medicine Systems Pharmacology (TCMSP) database and analysis platform (<https://old.tcmsp-e.com/tcmsp.php>) ^13^. Active components-target network was constructed with Cytoscape (V3.8.2)^14^.

**Anti-angiogenic mechanism and therapeutic actions**

To identify molecular mechanism of therapeutic actions and anti-angiogenic effect of CHM formulae, functional enrichment analysis performed by using Metascape (<https://metascape.org/>) ^15^. Functional enrichment analysis was performed with Gene Ontology database and KEGG database. DEGs related to angiogenesis, which identified from Gene Ontology database (GO term: 0001525), was analyzed for mechanism of anti-angiogenic effect of CHM formulae.

**Quantitative PCR**

Total RNA of endometriotic lesions were extracted with Qiagen RNeasy Plus Universal Mini Kit and reverse-transcripted with Takara PrimeScript RT reagent Kit. Gene expression was quantified by using TB Green Premix Ex Taq Kit with primers shown in supplementary table S2.

**Statistical analysis**

Quantitative data were shown as mean±SEM. The differences between treatment groups and control groups were evaluated by One Way ANOVA followed by Dunnett's multiple post-hoc comparisons. p<0.05 was considered as significant.

**Supplementary Figs**


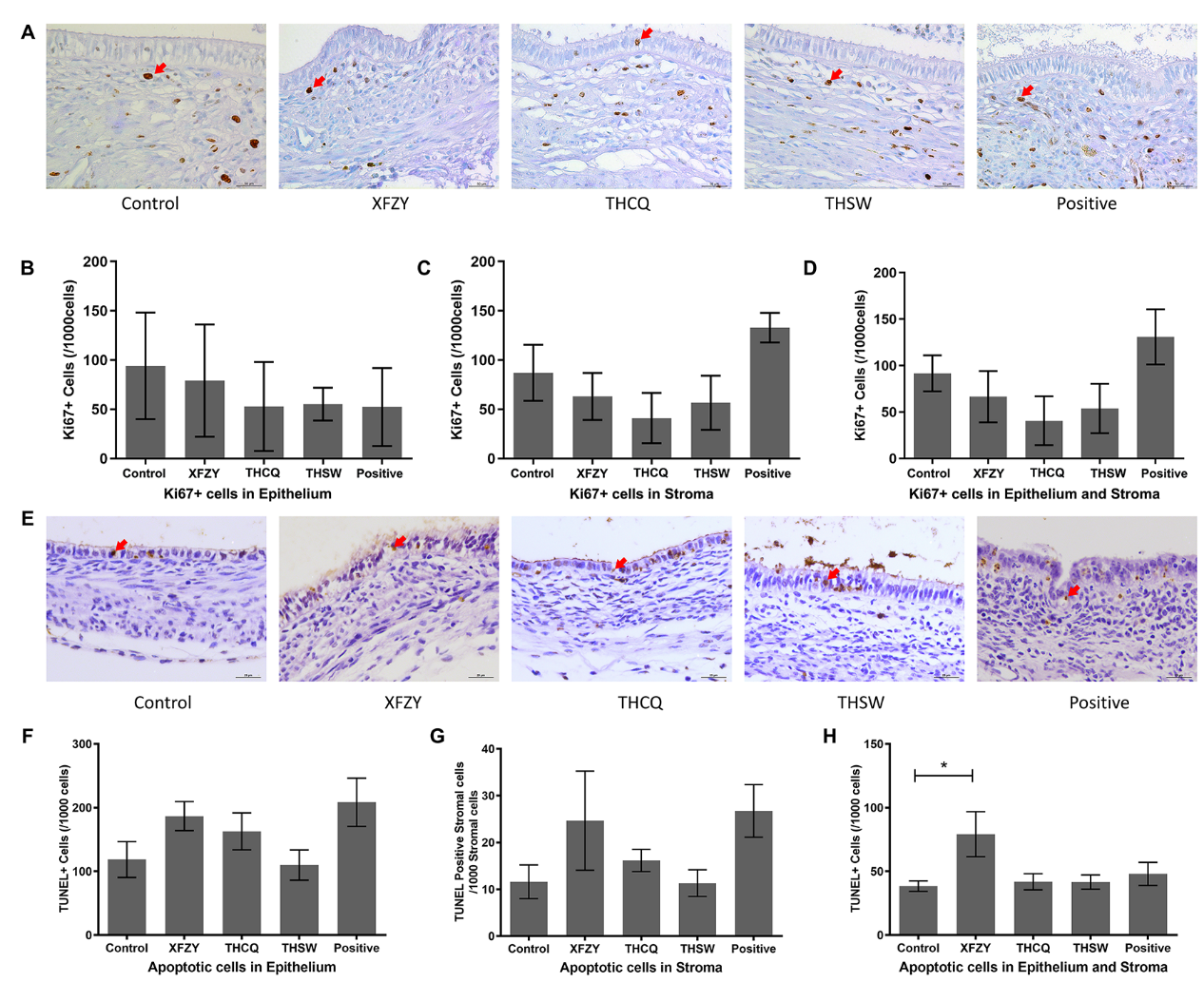


**Fig. S1,** Anti-Proliferative effect, and apoptotic effect of effective CHM formulae. **A,** Ki67 staining of ectopic lesions, representative image were shown; Red arrow: indicating of representative staining results; Scar bar=25μm. **B,** Proliferation in epithelium of ectopic lesions was assessed by the positive staining per 1000 cells in 5 random views. **C,** Proliferation in stroma of ectopic lesions was assessed by the positive staining per 1000 cells in 5 random views. **D,** Proliferation in both epithelium and stroma of ectopic lesions was assessed by the positive staining per 1000 cells in 5 random views. **E,** Tunel assay of ectopic lesions, representative image were shown; Red arrow: indicating of representative staining results; Scar bar=25μm*.* **F,** Apoptosis in epithelium of ectopic lesions was assessed by the positive staining per 1000 cells in 5 random views. **G,** Apoptosis in stroma of ectopic lesions was assessed by the positive staining per 1000 cells in 5 random views. **H,** Apoptosis in both epithelium and stroma of ectopic lesions was assessed by the positive staining per 1000 cells in 5 random views. Data are shown as mean±SEM, n=4 for control group and n=3 for CHM formulae group; *: p<0.05.


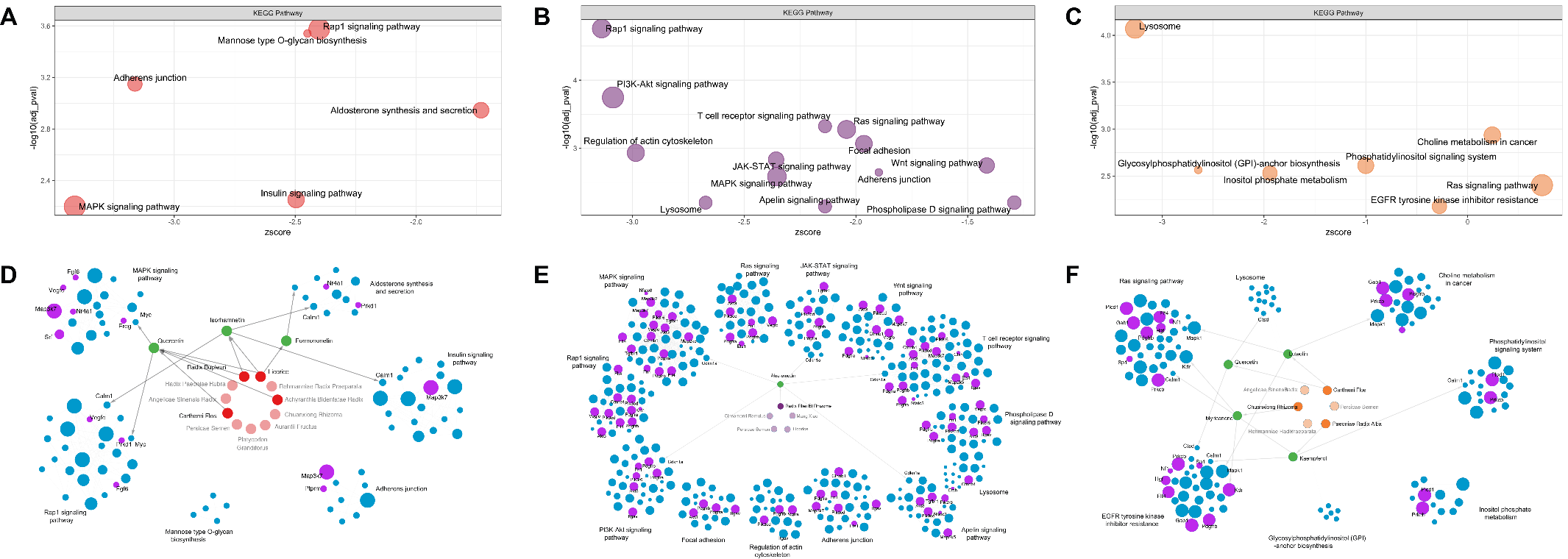


**Fig. S2**, Anti-endometriosis therapeutic actions of CHM formulae.**A, B, and C**: Functional enrichment of differential expression genes based on Kegg database; **D, E, and F**: PPI network of herbs, active components and differential expression genes enriched in Kegg pathways; Red, purple and yellow circles: herbs; Green circles: active components; blue circles: differential expression genes enriched in Kegg pathways. Deep Purple: differential expression genes related to angiogenesis.


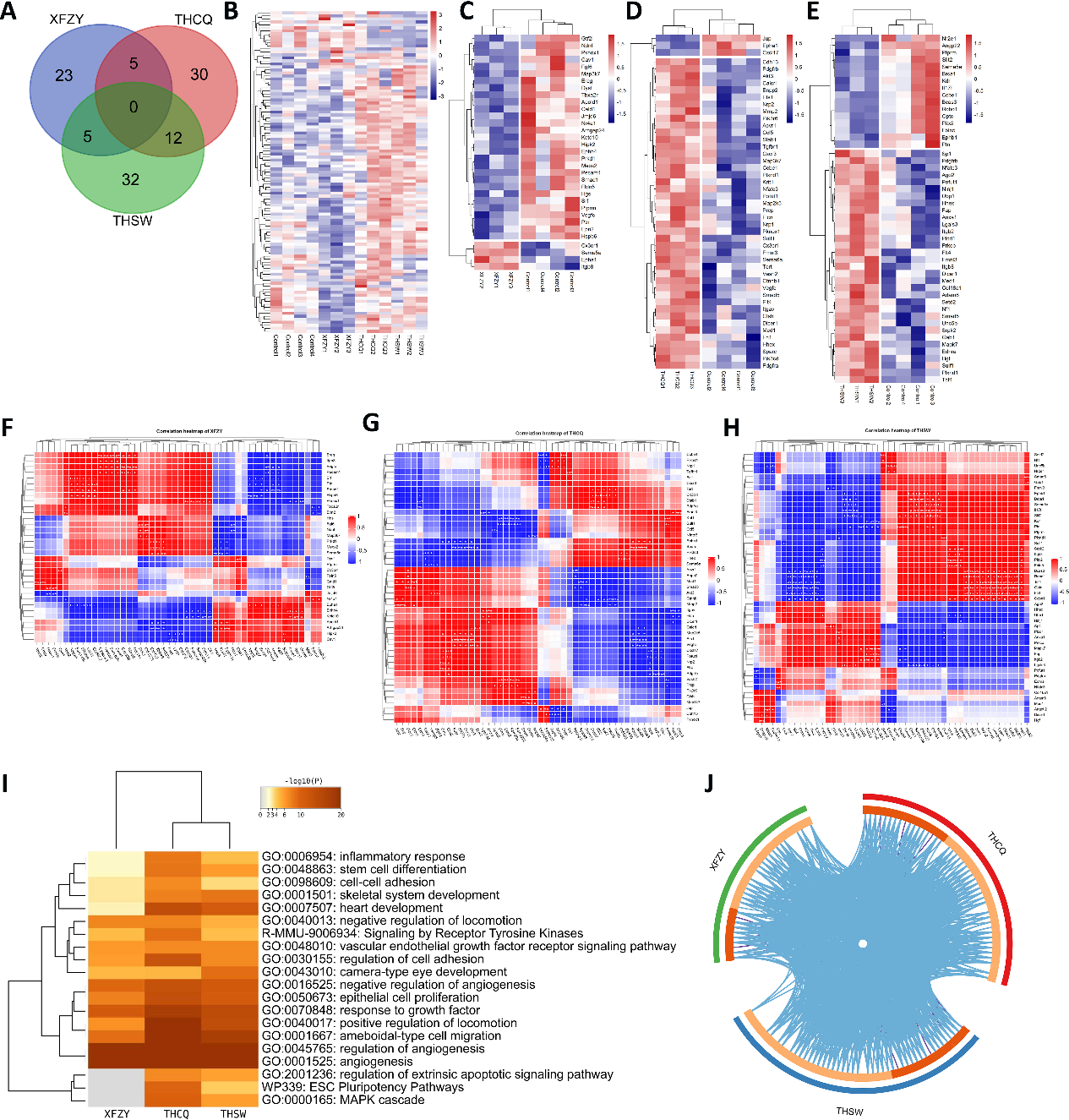


**Fig. S3**, Anti-angiogenic effect of CHM formulae in endometriosis. **A**, Venn diagram of differential expression angiogenic genes from 3 formulae; **B**, expression profile of differential expression angiogenic genes from 3 formulae; **C**, expression profile of differential expression angiogenic genes from lesions treated with XFZY; **D**, expression profile of differential expression angiogenic genes from lesions treated with THCQ; **E**, expression profile of differential expression angiogenic genes from lesions treated with THSW; **F, G, and H,** Pearson correlation heatmap of differential expressed genes and angiogenesis genes for XFZY, THCQ and THSW, respectively. Red indicates high positive correlation and blue is high negative correlation; I **and J**, functional enrichment analysis of differential expression angiogenic genes from 3 formulae;


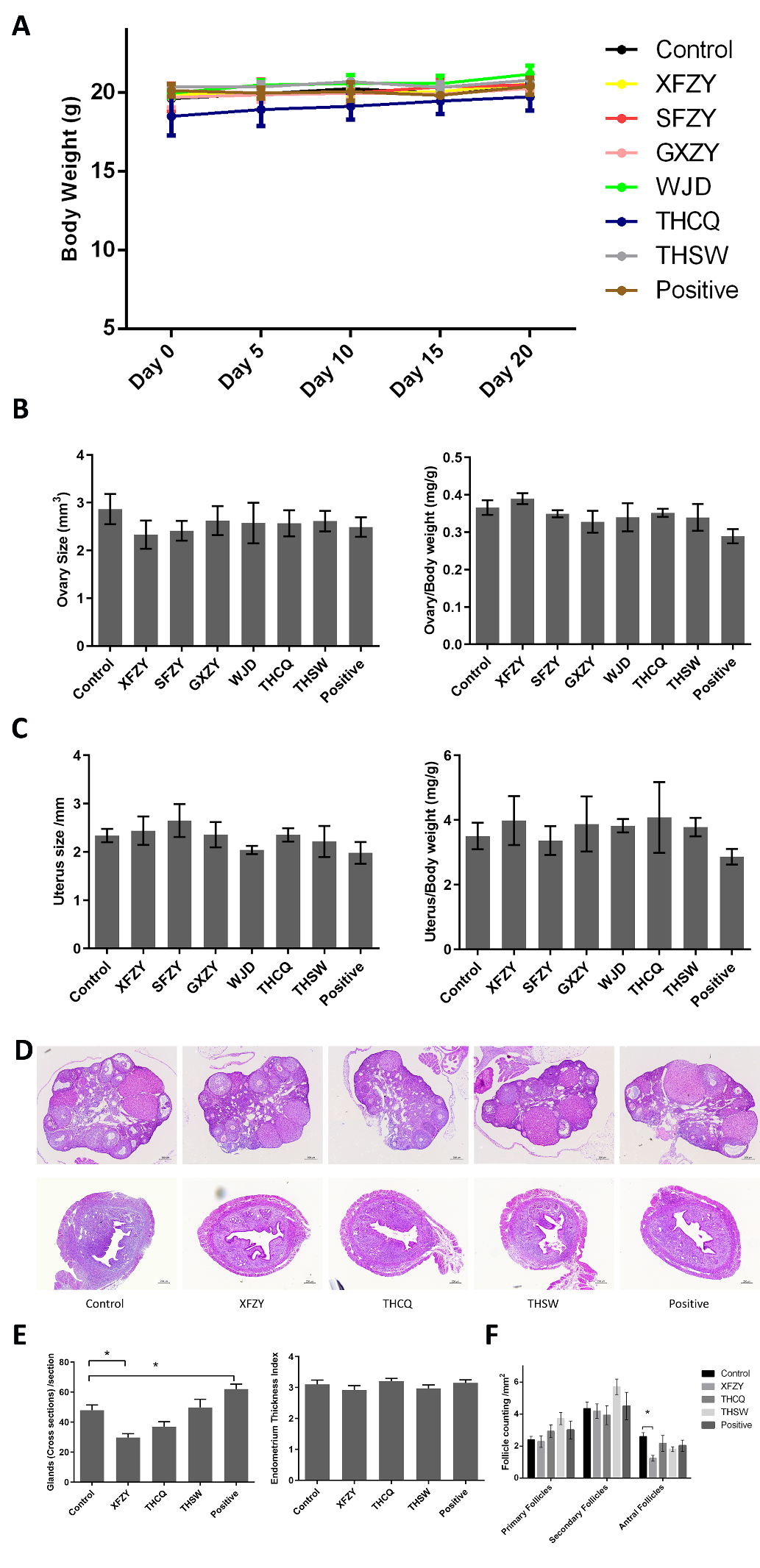


**Fig. S4**, Safety of CHM formula and individual herbs. **A,** Body weight change during CHM formulae intervention. **B**, Effect of different CHM formulae on weight and size of ovary after treatment with CHM formulae. **C**, Effect of different CHM formulae on weight and size of uterus. **D**, Histological change of uterus and ovary after treatment with negative control, CHM formulae and positive control, *Scar bar: 200um*. **E**, Uterine gland count and endometrium thickness index after treatment with negative control, CHM formulae and positive control. **F**, Follicle count after treatment with different CHM formulae. Data are shown as mean±SEM, n=4 for control group and n=3 for CHM formulae group ; *: p<0.05

Supplementary table S1 commonly used CHM formulae for endometriosis

| ID | Chinese Name | No. of  studies used^1^ | Frequency (%)^2^ | Ranking | Chinese  pharmacopoeia | CHM ancient  treatise | Potential Therapeutic effect^4^ | Potential application5 for endometriosis | Selected or not selected |
| --- | --- | --- | --- | --- | --- | --- | --- | --- | --- |
| 1 | Dan’e Fukang Jiangao (DEFK) | 42 | 18.18 | 1 | NO | NO | 1. Promoting blood circulation for removing blood stasis 2. Dispersing stagnated liver Qi 3. Regulating menstruation and pain relief 4. Softening and resolving masses | 1. Irregular menstruation 2. Dysmenorrhea 3. Menstrual discomfort 4. Lump accumulation | NO |
| 2 | Xuefu Zhuyu Tang  (XFZY) | 30 | 12.99 | 2 | YES | YES  (医林改错) | 1. Promoting blood circulation for removing blood stasis 2. Promoting qi circulation to relieve pain | 1. Chest blood stasis syndrome | YES |
| 3 | Neiyi Fang (NYF) | 24 | 10.39 | 3 | NO | NO | 1. Activating qi to resolve blood stasis 2. Softening and resolving masses | Endometriosis-associated symptoms | NO |
| 4 | Shaofu Zhuyu Tang  (SFZY) | 22 | 9.52 | 4 | YES | YES  (医林改错) | 1. Promoting blood circulation for removing blood stasis 2. Warming meridian to relieve pain | 1. Blood lump accumulation 2. Dysmenorrhea 3. Irregular menstruation 4. Menoxenia | YES |
| 5 | Wenjing Tang (WJD) | 20 | 8.66 | 5 | NO | YES  (金匮要略) | 1. Warming meridian and dispersing cold 2. Nourishing blood to remove stasis | 1. Thoroughfare and controlling vessel vacuity cold 2. Stagnation of blood stasis 3. Irregular menstruation 4. Coldness and pain in the lower abdomen | YES |
| 6 | Xiaoyi Fang (XYF) | 18 | 7.79 | 6 | NO | NO | NA | NA | NO |
| 7 | Gexia Zhuyu Tang  (GXZY) | 8 | 3.46 | 7 | NO | YES  (医林改错) | 1. Promoting blood circulation for removing blood stasis 2. Promoting qi circulation to relieve pain | 1. Stagnation Qi of diaphragm 2. Lump accumulation 3. Abdominal pendant 4. Unexplained pain | YES |
| 8 | Taohe Chengqi Tang  (THCQ) | 7 | 3.03 | 8 | NO | YES  (伤寒论) | 1. Breaking blood stasis | 1. Blood stasis 2. Amenorrhea 3. Dysmenorrhea 4. Lochiostasis | YES |
| 9 | Danggui Sini Tang  (DGSN) | 7 | 3.03 | 9 | NO | YES  (伤寒论) | 1. Nourishing blood to dissipate cold 2. Warming meridian to invigorate pulse-beat | 1. Typhoid fever 2. Sluggishness of blood circulation 3. Reversal cold of the extremities | NO |
| 10 | Eleng Jiaonang  (ELJN) | 6 | 2.60 | 10 | NO | NO | NA | NA | NO |
| 11 | Taohong Siwu Tang  (THSW) | 6 | 2.60 | 10 | NO | YES  (医宗金鉴) | 1. Removing stasis 2. Nourishing blood 3. Promoting Qi circulation | 1. Regulating qi and the blood 2. Irregular menstruation 3. Blood stasis | YES |

1: No. of studies used: total number of studies used this CHM formula based on literature search.

2: Frequency: frequency of studies used this CHM formula.

3: NA: not applicable.

4: Potential therapeutic effect and specific application for endometriosis were either extract from Chinese Pharmacopoeia or CHM ancient treatise

Supplementary table S2: Primers used in this study

| Genes | Forward primer | Reverse primer | Product size |
| --- | --- | --- | --- |
| Cav1 | ATGTCTGGGGGCAAATACGTG | CGCGTCATACACTTGCTTCT | 132 |
| Smad1 | GCTTCGTGAAGGGTTGGGG | CGGATGAAATAGGATTGTGGGG | 147 |
| Gtf2i | AGGCCCTTCTGAAACTGATGG | GGTCACCTCAACTTCGGGG | 172 |
| Srf | GGCCGCGTGAAGATCAAGAT | CACATGGCCTGTCTCACTGG | 159 |
| Ctnnb1 | ATGGAGCCGGACAGAAAAGC | CTTGCCACTCAGGGAAGGA | 108 |
| Tgfbr1 | TCTGCATTGCACTTATGCTGA | AAAGGGCGATCTAGTGATGGA | 100 |
| Ctsh | ACCGTGAACGCCATAGAAAAG | TGAGCAATTCTGAGGCTCTGA | 234 |
| Nrp | GACAAATGTGGCGGGACCATA | TGGATTAGCCATTCACACTTCTC | 104 |
| Gab1 | GAAGTTGAAGCGTTATGCGTG | AGAAAATCCGGTCGATGGTGT | 227 |
| Hgf | ATGTGGGGGACCAAACTTCTG | GGATGGCGACATGAAGCAG | 79 |
| Sp1 | GCCGCCTTTTCTCAGACTC | TTGGGTGACTCAATTCTGCTG | 131 |
| Kdr | TTTGGCAAATACAACCCTTCAGA | GCTCCAGTATCATTTCCAACCA | 177 |
| β-actin | GGCTGTATTCCCCTCCATCG | CCAGTTGGTAACAATGCCATGT | 154 |

**Supplementary References**

1. Bacci M, Capobianco A, Monno A, et al. Macrophages are alternatively activated in patients with endometriosis and required for growth and vascularization of lesions in a mouse model of disease. *Am J Pathol.* 2009;175(2):547-556.

2. Pelch KE, Sharpe-Timms KL, Nagel SC. Mouse model of surgically-induced endometriosis by auto-transplantation of uterine tissue. *J Vis Exp.* 2012(59):e3396.

3. Chinese Pharmacopoeia Commission. *Chinese Pharmacopoeia of the People's Republic of China* Vol 1. Bejing: China Medical Science and Technology Press; 2015.

4. Chinese Pharmacopoeia Commission. *Chinese Pharmacopoeia of the People's Republic of China.* Vol 1. Bejing: China Medical Science and Technology Press; 2010.

5. Rockville. Guidance for industry: estimating the maximum safe starting dose in initial clinical trials for therapeutics in adult healthy volunteers. *Center for Drug Evaluation and Research (CDER).* 2005.

6. Xu H, Lui WT, Chu CY, Ng PS, Wang CC, Rogers MS. Anti-angiogenic effects of green tea catechin on an experimental endometriosis mouse model. *Hum Reprod.* 2009;24(3):608-618.

7. Oliveira CR, Salvatori R, Nobrega LM, et al. Sizes of abdominal organs in adults with severe short stature due to severe, untreated, congenital GH deficiency caused by a homozygous mutation in the GHRH receptor gene. *Clin Endocrinol (Oxf).* 2008;69(1):153-158.

8. Cardiff RD, Miller CH, Munn RJ. Manual hematoxylin and eosin staining of mouse tissue sections. *Cold Spring Harb Protoc.* 2014;2014(6):655-658.

9. Schindelin J, Rueden CT, Hiner MC, Eliceiri KW. The ImageJ ecosystem: An open platform for biomedical image analysis. *Mol Reprod Dev.* 2015;82(7-8):518-529.

10. Rossi AG, Soares JM, Jr., Motta EL, et al. Metoclopramide-induced hyperprolactinemia affects mouse endometrial morphology. *Gynecol Obstet Invest.* 2002;54(4):185-190.

11. Bankhead P, Loughrey MB, Fernandez JA, et al. QuPath: Open source software for digital pathology image analysis. *Sci Rep.* 2017;7(1):16878.

12. Szklarczyk D, Gable AL, Lyon D, et al. STRING v11: protein-protein association networks with increased coverage, supporting functional discovery in genome-wide experimental datasets. *Nucleic Acids Res.* 2019;47(D1):D607-D613.

13. Ru J, Li P, Wang J, et al. TCMSP: a database of systems pharmacology for drug discovery from herbal medicines. *J Cheminform.* 2014;6:13.

14. Otasek D, Morris JH, Boucas J, Pico AR, Demchak B. Cytoscape Automation: empowering workflow-based network analysis. *Genome Biol.* 2019;20(1):185.

15. Zhou Y, Zhou B, Pache L, et al. Metascape provides a biologist-oriented resource for the analysis of systems-level datasets. *Nat Commun.* 2019;10(1):1523.
