# Supplementary figures and images for: Unique anti-angiogenic effects, pharmacological targets and therapeutic mechanisms of Chinese herbal medicines for endometriosis
Source: Genes Dis. 2023 Nov 11;11(5):101166. doi: 10.1016/j.gendis.2023.101166 (PMC11177055; doi:10.1016/j.gendis.2023.101166)

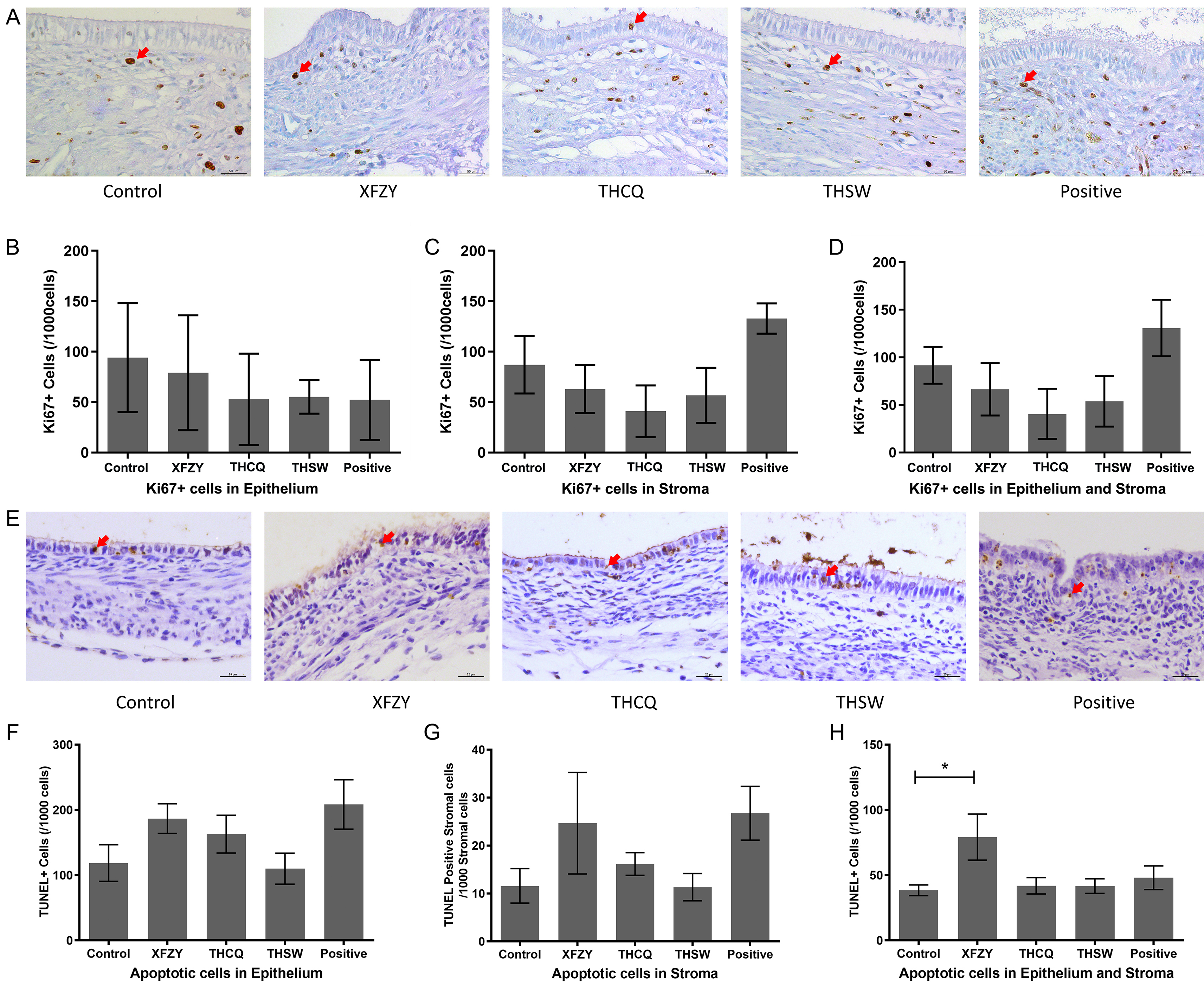

Supplement: Fig s1 [file figs1.jpg]

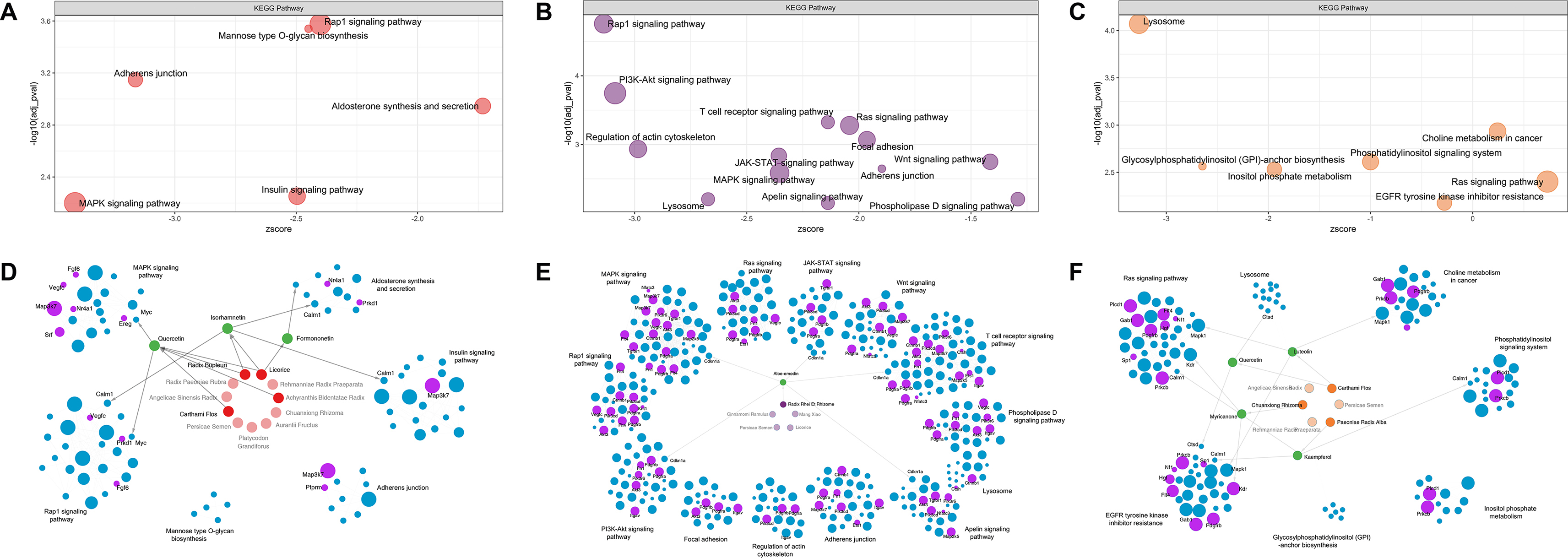

Supplement: Fig s2 [file figs2.jpg]

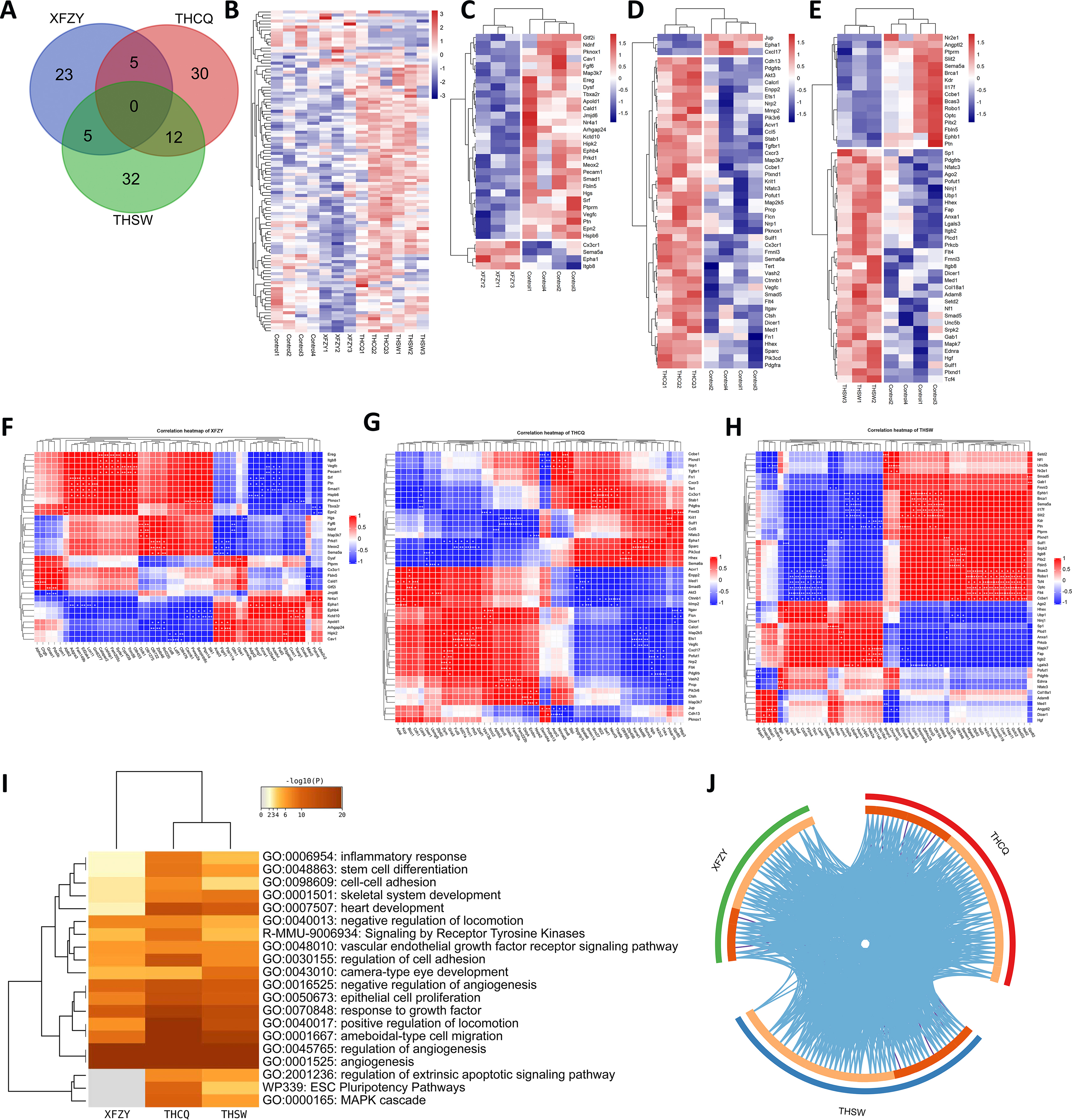

Supplement: Fig s3 [file figs3.jpg]

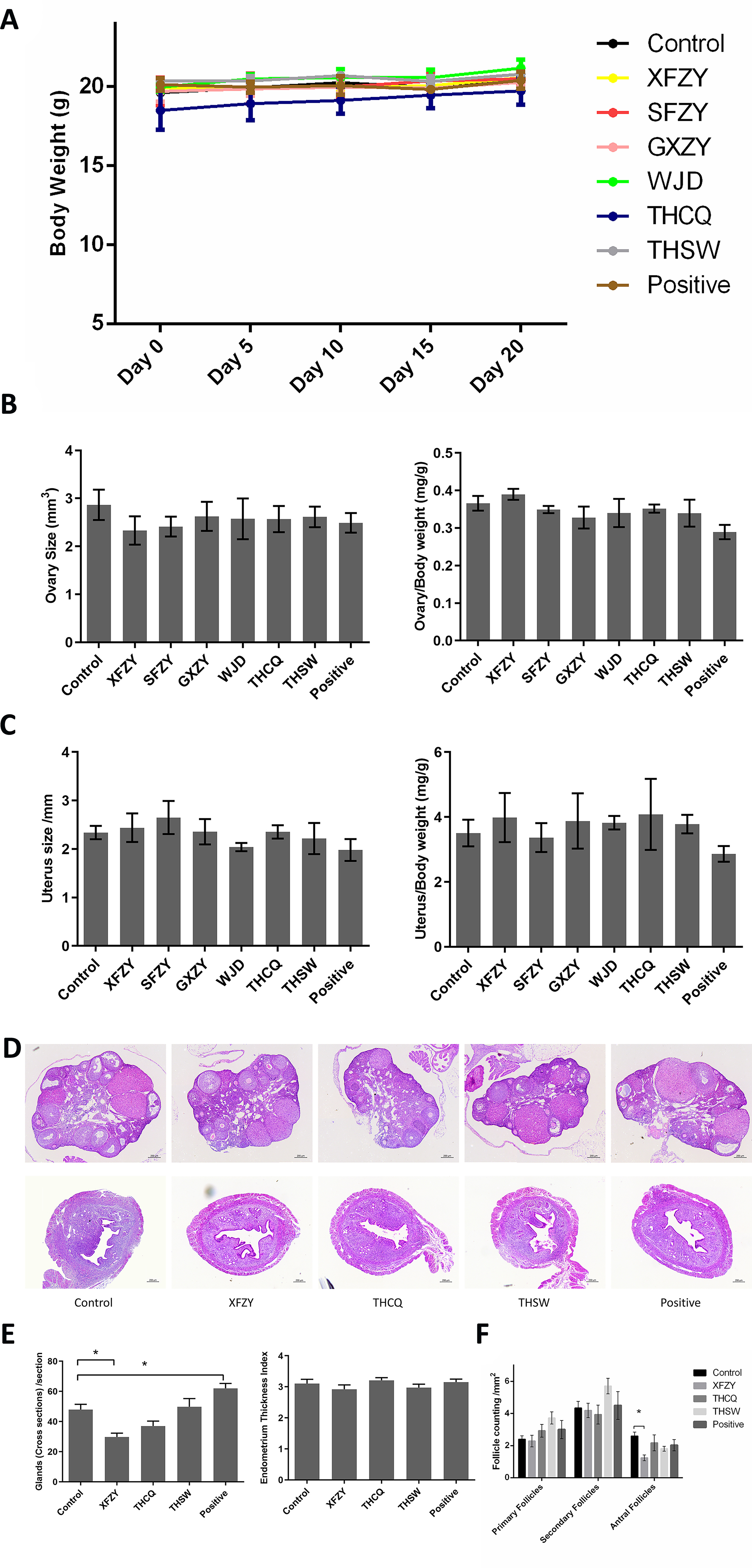

Supplement: Fig s4 [file figs4.jpg]
